# Psoriasis comorbidities in Germany: A population-based study on spatiotemporal variations

**DOI:** 10.1371/journal.pone.0265741

**Published:** 2022-03-22

**Authors:** Jobst Augustin, Sandra Wolf, Brigitte Stephan, Matthias Augustin, Valerie Andrees

**Affiliations:** Institute for Health Services Research in Dermatology and Nursing (IVDP), University Medical Center Hamburg-Eppendorf (UKE), Hamburg, Germany; Universita degli Studi di Roma Tor Vergata, ITALY

## Abstract

Psoriasis is a chronic disease with high impact on patients’ health and their quality of life. Psoriasis often occurs along with other comorbidities, but it is not yet clear what role the comorbidities play in regional psoriasis prevalence. This study investigates the temporal and regional variation of the psoriasis comorbidities diabetes mellitus type II, obesity, hypertension, affective disorders in Germany and their association with psoriasis prevalence. This analysis based on the population set of ambulatory claims data (2010–2017) of the statutory health insurance (SHI) in Germany (approx. 70.3 million people in 2017). Psoriasis comorbidities rates were determined on county level. We performed descriptive spatiotemporal analyses of psoriasis comorbidity prevalence rates. In addition, we identified and compared spatial clusters and examined regional variations using spatial statistical methods. The results show strong regional variations (northeast to south gradient) and an increasing psoriasis prevalence (max. 28.8%) within the observation period. Considering the comorbidities, results indicate comparable spatial prevalence patterns for diabetes mellitus type II, obesity and hypertension. This means that the highest prevalence of comorbidities tends to be found where the psoriasis prevalence is highest. The spatiotemporal cluster analyses could once again confirm the results. An exception to this is to be found in the case of affective disorders with different spatial patterns. The results of the studies show the first spatiotemporal association between psoriasis prevalence and comorbidities in Germany. The causalities must be investigated in more detail in order to be able to derive measures for improved care.

## Introduction

Psoriasis is a common chronic, relapsing, immune-mediated inflammatory disease with high impact on patients’ health and quality of life [1, 2]. This multifactorial chronic disorder essentially derives from the alteration of several signalling pathways and the co‐occurrence of genetic, epigenetic and nongenetic susceptibility factors [3]. Typical signs are thick, scaly pruritic plaques of the skin which show different typical patterns in distribution leading to a classification of several subtypes. Recent studies target genomic differences of patients with psoriasis to understand this inflammation and to explain different responses to treatment. This might lead to personalised therapy with the future identification of biomarkers [4]. However, the results are still controversial and more research in polygenics is crucial. Psoriasis and its interrelation with comorbidities is an interplay of genetic, immunologic, lifestyle, and environmental factors. Although psoriasis disease is a complex inflammatory systemic process, not limited to skin only, for the sake of simplicity, however, the short form ‘psoriasis’ is used in this study.

The prevalence of psoriasis ranges between 0.51% and 11.43% worldwide [5]. There are strong regional variations with lowest rates in the equator region [6, 7]. These variations can also occur on a small scale within a country [8]. Knowledge about the regional variations of psoriasis and its comorbidities in Germany is very limited and there are practically no small-scale studies on this.

Psoriasis often occurs with serious comorbidities, such as cardiovascular diseases, metabolic syndrome or depression [2, 9–11]. About a fifth of all patients with psoriasis develop inflammatory joint involvement. More and more, psoriasis is seen as a systemic inflammation nowadays which has a significant impact on the choice of therapy. In Germany, the overall prevalence of diabetes type II was about 7.3% in 2010 [12]. Obesity affected 18.1% in 2014/2015 of the German population [13]. Hypertension even affected 31.8% of the total population at that time [14]. For depressive disorders, a population-based study detected a one-year prevalence rate of 15.7% in 2017 [15]. All of these diseases have shown rising incidence and prevalence rates for Germany over the last years.

It is finally unclear whether psoriasis is related to the comorbidities, or the comorbidities are related to psoriasis and how they influence each other [16, 17]. Nevertheless, the burden of comorbidities is high. A review by Takeshita and colleagues on the epidemiology of psoriasis comorbidities found pooled odds ratios of 1.66 (95% confidence interval [CI] 1.46; 1.89) for psoriasis and obesity, 1.58 (95% CI 1.42; 1.76) for psoriasis and hypertension and 1.27 (95% CI 1.16; 1.40) for psoriasis and diabetes. For mood disorders, they found a hazard ratio of 1.39 (95% CI 1.37; 1.41) for depression. These associations are higher in more severe psoriasis cases [17]. Relevant comorbidities can have a significant impact on psoriasis care and should be addressed in routine care. In particular as trigger mechanism and stress factor, the knowledge about inflammatory pathogenetics is crucial for effective treatment strategies [18–20].

There are also indications of regional differences in the prevalence of hypertension [14, 21], diabetes type II [22], obesity [13] and depression [15] within Germany. Prevalence rates are often characterised by spatial patterns with differences between East and West Germany. Higher prevalence rates are mainly found in regions within East Germany that belong to the former German Democratic Republic. The unequal living conditions at that time are still evident today in the form of higher social deprivation in most of these regions [23].

To ensure optimal care for patients with psoriasis in Germany, it is important not only to know how psoriasis prevalence is regionally distributed but also how the prevalence of comorbidities is distributed. Since therapy of psoriasis is significantly impacted by comorbidities, they need to be considered in psoriasis treatment. Knowing regional variations of those comorbidities can help decision makers and medical service providers to distribute limited resources of healthcare supply sensibly and reasonably. It may also help to understand the aetiology and interrelations of psoriasis and its comorbidities more profoundly. To our knowledge, no study yet examined the temporal and regional variations and associations between comorbidities and psoriasis prevalence rates. The present study examines spatiotemporal variations of the prevalence rates of psoriasis comorbidities diabetes mellitus type II, obesity, hypertension, and affective disorders in Germany and their association with psoriasis prevalence.

## Materials and methods

### Data set and data preparation

German nationwide ambulatory claims data were provided by the National Association of Statutory Health Insurance Physicians (KBV) for the analyses. Almost 90% of the German population (approx. 70.3 million people) is covered by statutory health insurance (SHI) and represented in this data set. The data set contains information on billed diagnoses according to ICD-10 for psoriasis (L.40.-; except for L40.5+) and its comorbidities diabetes mellitus type II (E11.-), obesity (E66.0), hypertension (I10 to I15) and affective disorders (F30 to F39) from 2010 to 2017. To avoid overestimation of prevalence due to misdiagnosis, we defined cases as at least two billed confirmed diagnoses of psoriasis in different quarters within the year (M2Q criteria). For analyses of prevalence rates of chronic diseases in routine data, it is recommended to define cases that have a billed diagnosis in more than one quarter [24]. The regional reference of patients is based on their place of residence. The data of prevalence rates are direct sex- and age-standardised. The underlying population for standardisation was the statutory health insured population of the respected year with at least one medical contact. These are about 90% of the insured population.

### Descriptive and temporal trend analyses

Descriptive analyses were performed for the year 2017, and for the prevalence rate differences between 2010 and 2017. Spatiotemporal cluster analyses were conducted for the period from 2010 to 2017. To visualise the regional variation of the psoriasis prevalence, we used the standardised rates and subjected them to a spatial statistical smoothing method. Here, we applied a Bayesian smoothing model [25] that includes the underlying assumption that neighbouring regions have similar characteristics. With the model, rates of neighbouring counties are taken into account for the calculation of the smoothed rates. This leads to more stable data, taking spatial dependencies into account. First expected values were calculated for each county, indicating the prevalence that we would expect in accordance with the sex and age composition of the respective county. For example, a smoothed value for a county of 1.6 indicates that the observed rate is 1.6 times higher than the expected value in this county. In this way, random variations can be excluded, and spatial structures can be emphasised [26].

To quantify regional variations of the prevalence rates, first the extremal quotient (EQ) [27], Gini coefficient [28] and Moran’s I [29] were calculated. The EQ results from the division of the maximum identified value by the minimum value and therefore gives an expression of the amount of the regional variations [26]. For instance, an EQ of 3.1 means that the maximum value (county ’a’) is 3.1 times greater than the minimum value (county ‘b’). To avoid the influence of outliers, only data within the 1% and 99% percentile were used. Beside this, we used the Gini coefficient as a further statistical measure to describe the regional variation. The Gini coefficient measures the inequality between the values of a frequency distribution (here prevalence at county level). A Gini coefficient of 0 is an expression of complete homogeneity between the counties, meaning that all prevalence rates are equal. A Gini coefficient of 1 describes the maximum inequality between county values.

The spatial autocorrelation was measured with Moran’s I. One speaks of spatial autocorrelation when the presence of a specific characteristic (e.g., prevalence) in one area (e.g., county) makes its presence in a neighbouring area more or less likely [30] and a systematic pattern in the spatial distribution of a variable is present. A distinction is made between positive and negative autocorrelation: A positive one means that neighbouring or nearby areas are more similar; a negative one describes patterns in which neighbouring areas are dissimilar. Random patterns have no spatial autocorrelation. Moran’s I can take positive or negative values between 0 (random, no autocorrelation) and ±1 (dispersed/clustered, auto correlated) [29]. Beside spatial aspects, we analysed the association between psoriasis and its comorbidities over time. Here, we calculated a ratio on county level for each year: psoriasis with comorbidity divided with the comorbidity for each of the four comorbidities under examination. As there are less cases of psoriasis with the comorbidity than of the comorbidity, all ratios need to be < 1. Lower rates indicate fewer psoriasis patients in the group of comorbidity patients. To compare the development over time for each comorbidity, we used bee swarm diagrams. Analysis was conducted in R (R Core Team, Vienna, Austria) and figures were produced using the package ‘beeswarm’ [31].

### Spatiotemporal cluster analyses

To identify regional variations and their clusters in the prevalence rates of psoriasis comorbidities’, spatiotemporal cluster analyses were conducted. First, for all counties and each comorbidity, centroids were created in a geographic information system (GIS). GIS are software tools for the management, analysis and visualisation of spatial data respectively geodata. In this case, the data were processed with a GIS in order to be able to carry out the cluster analysis (here, preparing the data and creating the centroids). The clusters itself were computed with the software tool SaTScan. The method used is Kulldorf’s spatial scan statistic, which can be used to identify statistically significant, spatially compact clusters [32]. In this method, elliptical or circular windows move over the observation area and the observed values are compared with values expected under the null hypothesis. These follow a certain probability distribution. In each window corresponding to a possible cluster, the observed and expected cases and controls are contrasted and relative risks (RR) are calculated. Likelihood ratio statistics are used to identify the most likely clusters and assign a p-value. It is possible to scan for both high and low risk clusters [33].

The maximum spatial cluster size is defined by the percent of the population at risk, which bases on the Gini coefficient. With the Gini coefficient, among others, the degree of the heterogeneity within the clusters can be evaluated. It can be determined when it is preferable to report a collection of smaller clusters or a single large cluster containing the small clusters [34]. To run spatiotemporal cluster analyses, an underlying probability model must be chosen. Here, the selection of a model is based on the number of cases. The Bernoulli model is more suitable for higher numbers of cases than the Poisson model [32]. Due to the different numbers of cases and prevalence rates, we used the Bernoulli model for hypertension and the Poisson model for diabetes, obesity and affective disorders. For the Bernoulli model, we defined the cases as the number of patients with psoriasis with hypertension and the controls as the number of patients with psoriasis minus the number of hypertensives. In the Poisson model, the cases were defined as the number of patients with psoriasis with one of each comorbidity. Table 1 summarises the comorbidities, used Gini coefficients, population at risk and the used model.

**Table 1:** Used Gini coefficients, percentages of the population at risk and probability models.

Comorbidity	Optimal Gini coefficient	Population at risk (%)	Probability model
Hypertension	0.514	12	Bernoulli
Diabetes	0.072	15	Poisson
Obesity	0.074	20	Poisson
Affective disorders	0.084	4	Poisson

The spatial analyses based on the Euclidean distance using longitude and latitude. Other distance models would not have added value in the context of this study. The analyses were conducted on county level (N = 402 counties). The analyses were performed with ArcGIS 10.3.1 (ESRI Inc., Redlands, CA, USA), QGIS 3.10.4 A Coruna (QGIS Development Team), SaTScan v.9.6 (Boston, MA, USA) and R Core Team [35].

The ambulatory claims data had to be applied for at the National Association of Statutory Health Insurance Physicians (KBV). There, the data were prepared and made available. Due to data security, they were spatiotemporally aggregated on county level, therefore tracing back to individuals is not possible. Data users do not have access to the original data. As far as known, the data were not requested and analysed by other researchers.

## Results

### Descriptive spatial and temporal trend analyses

In 2017, the total number of psoriasis cases was N = 1,220,188 with an M2Q case definition. Slightly less men (48.3%) were observed. On county level, the mean standardised prevalence rate increased from 147.4 per 10,000 in 2010 to 173.5 in 2017.

**Fig 1:**
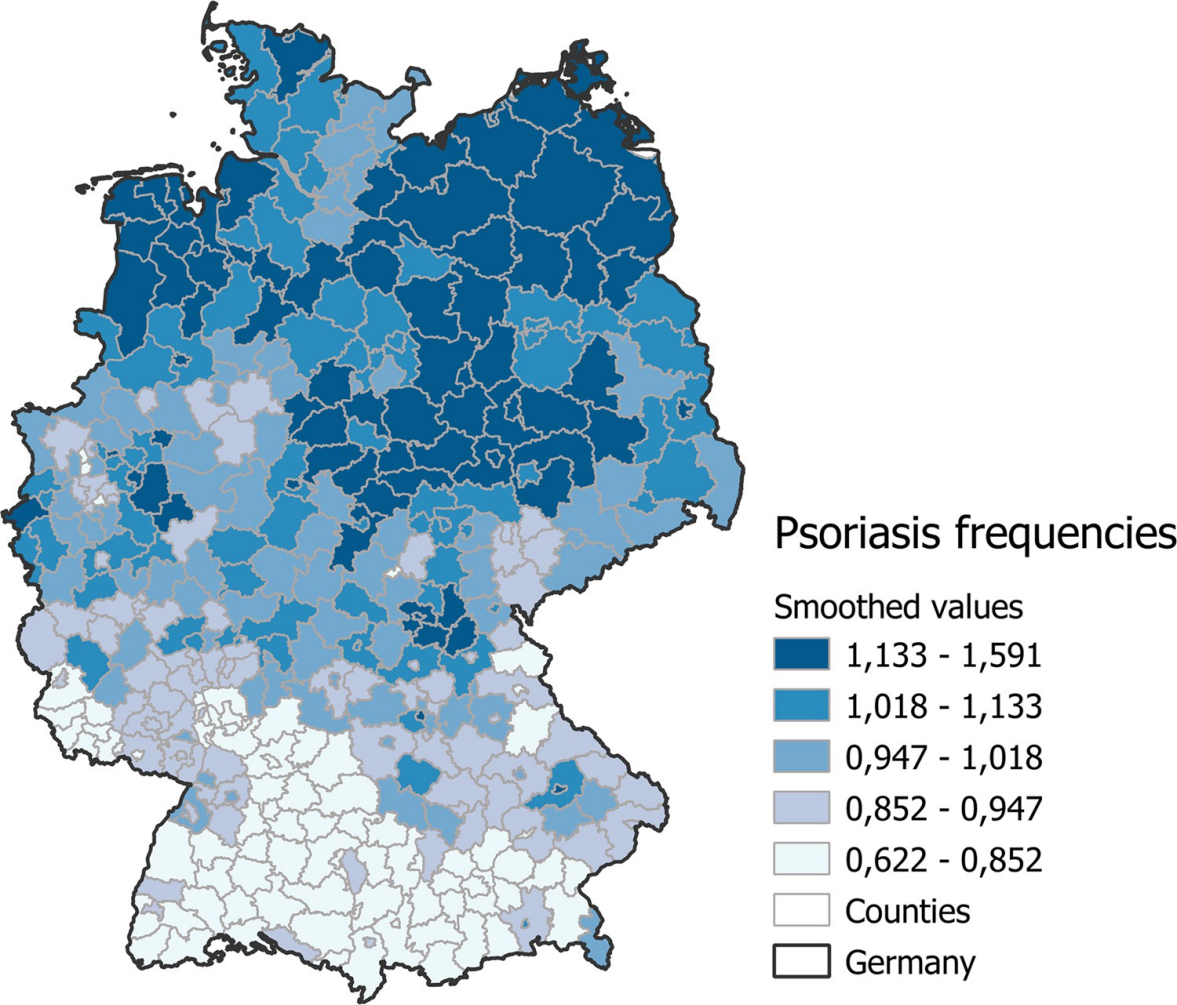
Smoothed regional patterns of the sex- and age-standardised overall psoriasis prevalence (regardless the factor comorbidity) in 2017 at the level of the 402 counties in Germany. Republished from Andrees et al. [36] under a CC BY-NC 4.0 license.

Fig 1 shows the smoothed regional variation of the age- and sex-standardised psoriasis prevalence in 2017. The map indicates a marked north-south gradient of psoriasis frequency. Spatial patterns with higher prevalence rates were identified in parts of North (-eastern) Germany, lower prevalence rates in Southern Germany. For all counties, we found an EQ of 2.48 and a Gini coefficient of 0.118. Moran’s I of the psoriasis prevalence is 0.47. This indicates a moderate positive spatial autocorrelation.

Considering comorbidity, we found the lowest prevalence rates for patients suffering from psoriasis and obesity together, followed by psoriasis combined with affective disorders as well as psoriasis with diabetes. The highest prevalence by far was found for patients with psoriasis and hypertension.

The strongest regional variation shows obesity (EQ 6.71, Gini coefficient 0.229), but the lowest spatial autocorrelation (Moran’s I 0.375). With a Moran’s I of 0.51, hypertension shows the highest spatial autocorrelation (Table 2).

**Table 2:** Descriptive statistics for comorbidities with and without psoriasis for 2017 (patients per 10,000 statutory health insured people).

	**Psoriasis without comorbidities**				
Year 2017	Mean	173.50			
	Median	168.20			
	Min	93.80			
	Max	340.90			
	Standard deviation	37.06			
	Extremal quotient	2.48			
	Gini coefficient	0.12			
	Moran’s I	0.47			
	**Psoriasis with comorbidities**				
		Diabetes	Obesity	Affective disorders	Hypertension
	Mean	33.94	15.36	33.86	90.76
	Median	31.54	14.05	33.65	86.51
	Min	13.82	4.50	15.76	37.53
	Max	84.72	50.20	70.16	217.79
	Standard deviation	10.68	6.81	8.09	26.12
	Extremal quotient	3.80	6.71	3.01	3.33
	Gini coefficient	0.17	0.23	0.13	0.16
	Moran’s I	0.44	0.38	0.37	0.51
	**Comorbidities without psoriasis**				
		Diabetes	Obesity	Affective disorders	Hypertension
	Mean	910.64	422.19	1,143.57	2,814.90
	Median	889.61	403.67	1,124.27	2,763.95
	Min	619.18	206.98	751.10	1,840.79
	Max	1,419.01	1,147.06	1,963.13	3,758.70
	Standard deviation	151.96	127.45	168.34	361.40
	Extremal quotient	1.93	3.41	1.96	1.74
	Gini coefficient	0.094	0.16	0.08	0.07
	Moran’s I	0.43	0.34	0.35	0.58

Analysing patterns of these diseases alone, without psoriasis, we found similar patterns with the highest prevalence rates for hypertension and the lowest for obesity. In terms of spatial variation, we identified the highest variation for obesity (EQ 3.41, Gini coefficient 0.16) and the lowest for hypertension (E Q 1.74, Gini coefficient 0.072). Again, we could identify the highest Moran’s I (0.582) for hypertension (Table 2).

**Fig 2:**
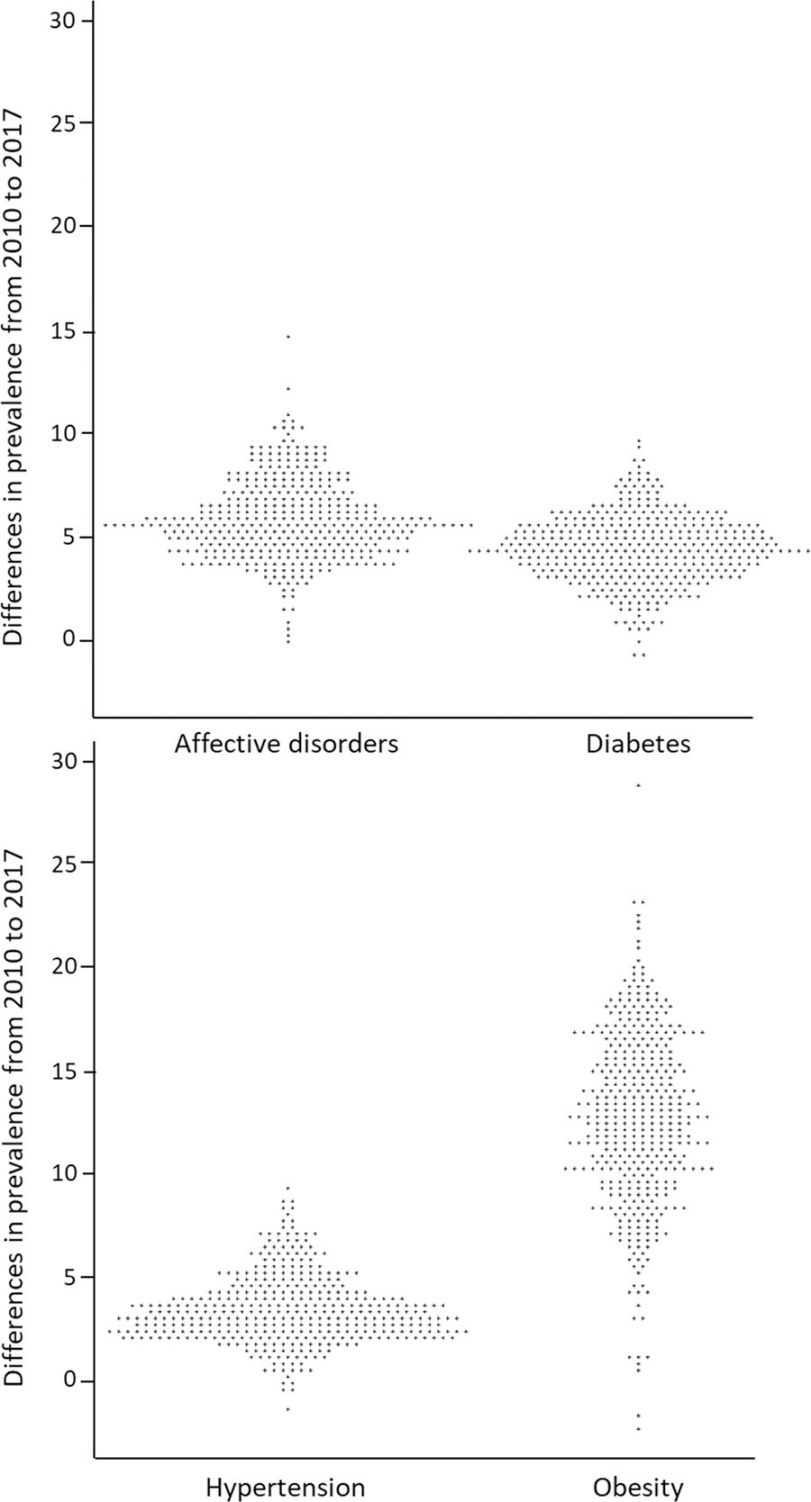
Bee swarm plots for the differences (cases per 10,000 statutory health insured people) in prevalence (psoriasis with comorbidities) between 2010 and 2017. Each point represents a county.

With regard to temporal aspects, we analysed the differences (cases per 10,000) in prevalence rates (psoriasis with comorbidities) between 2010 and 2017 and plotted them in a bee swarm diagram (Fig 2). Each point in the diagram represents one county in Germany. The extent of distribution for affective disorders, diabetes and hypertension looks more or less similar. The values are between -2.1 and +28.8. In most counties, we found increasing prevalence rates. Only in few counties, the prevalence decreased. The distribution of obesity differs markedly from the other comorbidities. Here, more counties were identified with stronger increasing prevalence rates.

**Fig 3:**
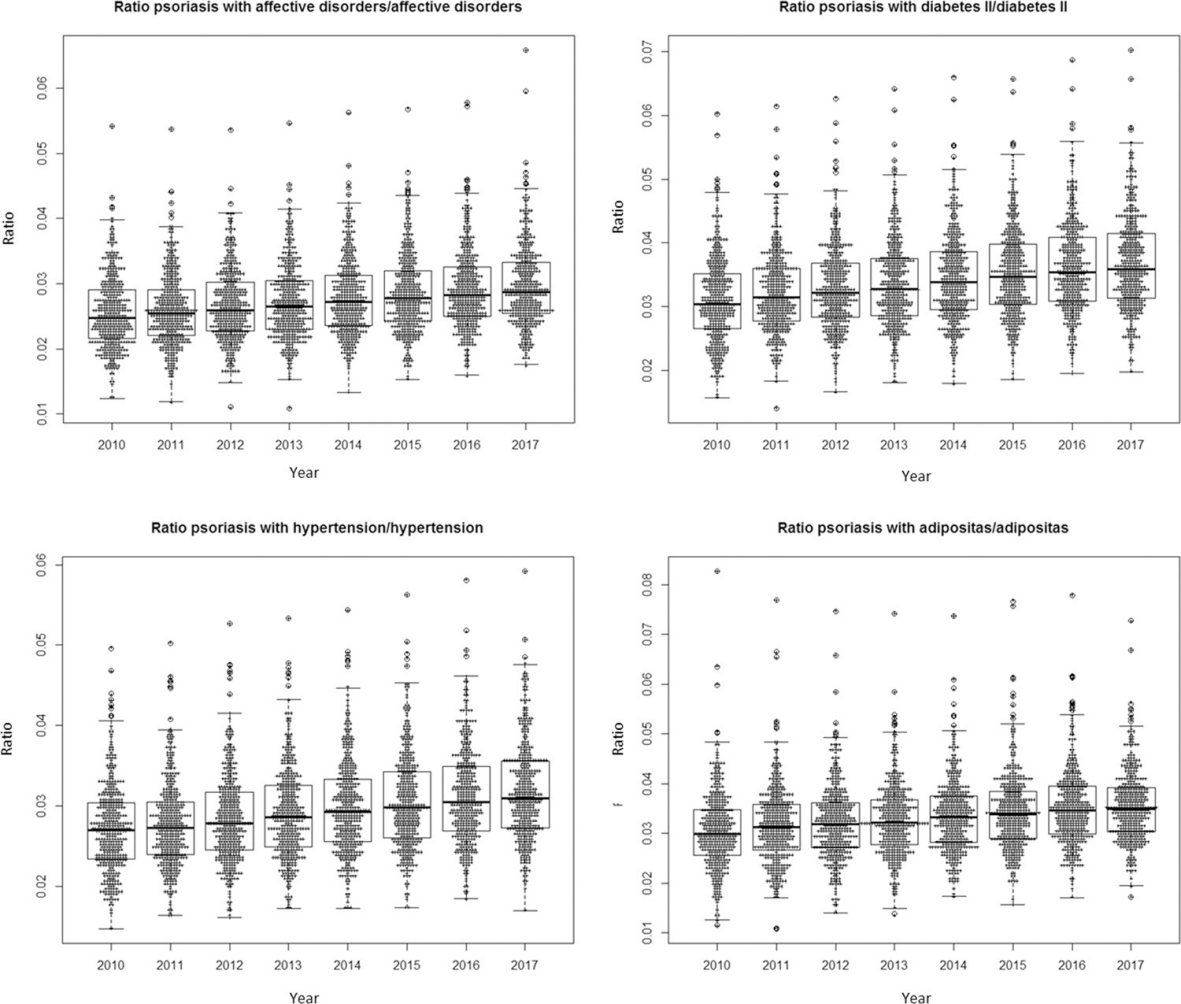
Bee swarm plots of ratios for psoriasis with comorbidity/comorbidity on county level. Each point represents one county.

Furthermore, we analysed the change (2010–2017) of the ratios (psoriasis with comorbidity divided by the comorbidity) on county level (Fig 3).

The results show rising values for each disease over time. In addition, the mean ratio for all diseases rose each year between 2010 and 2017; from 0.025 to 0.029 for affective disorders, from 0.031 to 0.037 for diabetes, from 0.027 to 0.032 in hypertension and from 0.031 to 0.035 in obesity.

### Spatiotemporal cluster analyses

**Fig 4:**
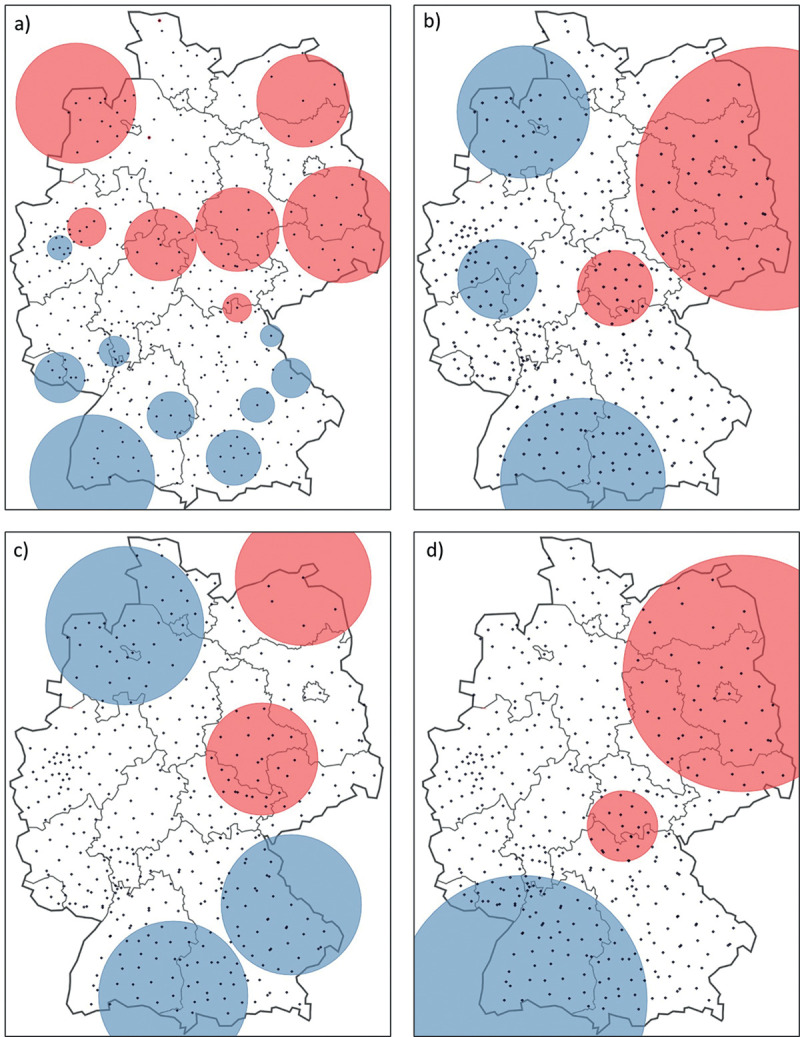
Spatiotemporal cluster (only significant clusters) analyses on county level for a) affective disorders, b) diabetes, c) hypertension and d) obesity (maps based on Andrees et al. [36]). High cluster = red, low clusters = blue.

The results of the spatiotemporal cluster analyses are displayed in Fig 4 and Table 3. For affective disorders (Fig 3A), we found in total 18 significant clusters, nine high clusters (high prevalences) and nine low clusters (low prevalences). High clusters were tendentially found in the northern part of Germany, low clusters in South Germany. All clusters are to be assigned to the period 2010/1/1–2017/12/31. The number of clusters for the other comorbidities, especially for diabetes and obesity, is much lower. We found five (two high and three low) significant clusters for diabetes (Fig 3B). Here, we identified the low clusters in West Germany and the two high clusters in East Germany. Cluster 5 is to be assigned to the period 2010/1/1–2013/12/31, the others to 2010/1/1–2017/12/31. Hypertension (Fig 3C) shows in total five significant clusters, two high and three low clusters. As with the other comorbidities, the high clusters are located mainly in East Germany. Both high clusters (1 and 4) are to be assigned to the period 2010/1/1–2017/12/31, the low clusters 2 and 4 as well. The low cluster 5 refers to the period 2010/1/1–2013/12/31. For obesity (Fig 3D), we found three significant clusters. One low cluster in South Germany, one high cluster in the middle and one in East Germany. Cluster 1 and 2 are associated with the period 2010/1/1–2017/12/31, cluster 3 with 2014/1/1–2017/12/31. In addition to that, Table 3 describes each cluster in detail with population (number of comorbidities), cases (number of psoriasis and comorbidity), expected cases (cases taking into account the null hypothesis), the ratio between observed and expected cases, the relative risk and p-value. The clusters are sorted by p-value. For instance, this means for affective disorders and cluster 1: between 2010/1/1–2017/12/31 we found n = 16,155 cases with affective disorders in this cluster and n = 4,747 cases with psoriasis and affective disorders. Considering the null hypotheses, we calculated n = 3,578 expected cases and a ratio between observed and expected cases of 1.33. The relative risk is 1.34, indicating that the relative risk for psoriasis and affective disorders in this cluster and period is 1.34 times higher than outside of this period and cluster.

**Table 3:** Results (only significant clusters) spatiotemporal cluster analyses in addition to Fig 4A–4D.

Comorbidity	Cluster (no.)	Type of cluster	Time frame	Population	No. of cases	Expected cases	Observed/expected	RR	p-value
Affective disorders (Fig 4A)	1	high	2010/1/1–2017/12/31	16,155	4,747	3,578	1.33	1.34	< 0.001
	2	high	2010/1/1–2017/12/31	14,785	4,275	3,275	1.31	1.32	< 0.001
	3	low	2010/1/1–2017/12/31	15,961	2,660	3,535	0.75	0.75	< 0.001
	4	low	2010/1/1–2017/12/31	16,109	2,709	3,568	0.76	0.75	< 0.001
	5	high	2010/1/1–2017/12/31	8,983	2,693	1,990	1.35	1.35	< 0.001
	6	low	2010/1/1–2017/12/31	13,315	2,358	2,949	0.80	0.79	< 0.001
	7	low	2010/1/1–2017/12/31	9,797	1,702	2,170	0.78	0.78	< 0.001
	8	low	2010/1/1–2017/12/31	14,489	2,661	3,209	0.83	0.82	< 0.001
	9	high	2010/1/1–2017/12/31	5,215	1,492	1,155	1.29	1.30	< 0.001
	10	high	2010/1/1–2017/12/31	14,462	3,727	3,203	1.16	1.17	< 0.001
	11	high	2010/1/1–2017/12/31	14,289	3,682	3,165	1.16	1.17	< 0.001
	12	high	2015/1/1–2017/12/31	937	176	87	2.02	2.02	< 0.001
	13	low	2010/1/1–2017/12/31	10,027	1,872	2,221	0.84	0.84	< 0.001
	14	high	2010/1/1–2017/12/31	14,348	3,603	3,178	1.13	1.14	< 0.001
	15	low	2010/1/1–2017/12/31	7,056	1,311	2,323	0.84	0.84	< 0.001
	16	low	2010/1/1–2017/12/31	4,433	802	982	0.82	0.82	< 0.001
	17	high	2010/1/1–2017/12/31	1,068	333	237	1.41	1.41	< 0.001
	18	low	2010/1/1–2017/12/31	3,592	657	795	0.83	0.82	0.031
Diabetes (Fig 4B)	1	high	2010/1/1–2017/12/31	9,722	18,144	14,457	1.25	1.31	< 0.001
	2	low	2010/1/1–2017/12/31	9,665	11,987	14,371	0.83	0.81	< 0.001
	3	low	2010/1/1–2017/12/31	8,463	11,171	12,584	0.89	0.87	< 0.001
	4	high	2010/1/1–2017/12/31	7,073	11,636	10,518	1.11	1.12	< 0.001
	5	low	2010/1/1–2013/12/31	8,163	5,039	5,851	0.86	0.85	< 0.001
Hypertension (Fig 4C)	1	high	2010/171–2017/12/31	92,263	64,844	61,756	1.05	1.06	< 0.001
	2	low	2010/1/1–2017/12/31	93,163	59,702	62,358	0.96	0.95	< 0.001
	3	high	2010/1/1–2017/12/31	37,737	26,282	25,259	1.04	1.04	< 0.001
	4	low	2010/1/1–2017/12/31	92,818	60,918	62,127	0.98	0.98	< 0.001
	5	low	2010/1/1–2013/12/31	29,307	18,965	19,617	0.97	0.97	< 0.001
Obesity (Fig 4D)	1	high	2010/1/1–2017/12/31	21,910	7,519	6,089	1.23	1.31	< 0.001
	2	low	2010/1/1–2017/12/31	21,729	4,920	6,038	0.81	0.78	< 0.001
	3	high	2014/1/1–2017/12/31	11,876	2,360	2,102	1.12	1.13	0.002

RR, relative risk.

## Discussion

To date, it is not clear what the association between psoriasis and psoriasis comorbidities is when spatial and temporal aspects are taken into account. Therefore, the aim of this study was to analyse the spatiotemporal association between psoriasis and their most common comorbidities (and also very frequent non-communicable disease in Germany). For this study we used regional ambulant billing data considering the M2Q criteria and analysed them with different (spatial-) statistical methods.

The analyses for psoriasis in Germany reveal a striking north-south gradient with higher prevalence rates in northern regions. A comparable north-south gradient was also found for the United Kingdom (UK) [8] and for worldwide prevalence rates in a review [7]. These differences might be influenced by the amount of regional UV radiation respectively sun duration, and its association with vitamin D metabolism, but several aspects are not yet conclusively understood. We focused on the aspect of possible influence of coexisting comorbidity with psoriasis as a promotor, which might also be spatially associated with the patterns of psoriasis prevalence rates.

### Regional variations of prevalence rates

With focus on regional variations, we found the strongest variation for obesity as a comorbidity. The analysis of the four selected comorbidities alone, without the restriction to patients with psoriasis, also shows the highest variations in obesity. Strong regional differences and the pattern of obesity mostly occurring in Northeastern Germany. We detected highest prevalence rates for women with obesity in Brandenburg and Mecklenburg-West Pomerania and for men in Mecklenburg-West Pomerania. The high obesity prevalence is probably associated with the social deprivation and thus lifestyle and health behaviour in these regions (the former German Democratic Republic). The increased prevalence of psoriasis in this region also fits into this pattern.

The main focus of this study was the spatiotemporal cluster analysis to identify eventually similar spatial patterns in psoriasis and comorbidity prevalence. A comparison of the comorbidity clusters with the psoriasis cluster shows that the highest prevalence of comorbidities is tendentially found where the psoriasis prevalence is also highest. An exception to this is to be found in the case of affective disorders. Among the comorbidities, two different kinds of cluster patterns were detected. For diabetes, obesity and hypertension, diseases associated with the metabolic syndrome, the spatial patterns were all very similar. This can be explained by the fact that they are closely associated with each other and frequently occur together [37–39]. They show low clusters in Western Germany and high clusters in Eastern and Northeastern Germany. Almost all clusters did not occur temporarily, but for all years constantly seen throughout 2010 to 2017. This means that these regional variations are very stable. Here, a major factor could be the more deprived areas of Eastern Germany [40], since other studies show that diseases of the metabolic syndrome occur more often in deprived areas [41, 42].

As already mentioned before, affective disorders show different patterns for Germany. Here, low clusters occur in the southern and high cluster in the [23] northwestern part of Germany. This seems a little bit more similar to the psoriasis clusters we detected for Germany. One possibility as to why affective disorders cluster differently from the other comorbidities, but similarly to psoriasis, could be that these regions are known to have a higher prevalence rate for patients with psoriasis. Beside this, the association between psoriasis and affective disorders is different from the other comorbidities. Psoriasis can act as a trigger for the onset of affective disorders. This is due to the high mental burden of stigma and appearance in psoriasis patients [43, 44]. While the other comorbidities (hypertension, diabetes and obesity) can act as triggers for the onset of psoriasis [45, 46].

The highest comorbidity prevalence was found for patients with psoriasis and hypertension, followed by diabetes, affective disorders and obesity. The prevalence of hypertension among psoriasis patients was about three times higher than for the other comorbidities. The sole consideration of comorbidities shows the highest prevalence in hypertension, followed by affective disorders, diabetes and overweight [2, 47, 48]. This is conclusive with observations of the coexistence of psoriasis as chronic systemic inflammation and the inflammatory axis in the development of cardiovascular disease as a consequence of metabolic changes. Patients with chronic psoriasis show higher levels of inflammatory biomarkers and evidence of insulin resistance driven by pro-inflammatory cytokines leading to the comorbidities in focus [49]. In addition, psoriasis can imply a psychological impairment, which can influence patient’s personality. This also applies to the so-called Type D personality, which is defined by the combination of social inhibition and negative affectivity. The Type D personality has been associated with impaired health-related quality of life (HRQoL) and increased cardiovascular risk. Both facts are associated with moderate to severe psoriasis [50].

All in all, the interpretation of the spatial clusters of psoriasis and its comorbidities is complex and leads to some hypotheses on how the clusters evolved. In addition to changing prevalence, the physicians billing behaviour may also be a relevant factor. We cannot distinguish this aspect in the data. Beside this, region-specific changes in the healthcare structure (e.g., settlement of new physicians) may also be responsible.

### Development of prevalence rates

We found that prevalence rates of psoriasis and all comorbidities were rising between 2010 and 2018. By calculating ratios for psoriasis with each comorbidity for each year, we found that the prevalence rate of psoriasis with comorbidities rises more than the rates of the comorbidities (Fig 3), as the ratios increased over the years. On the one hand, it could be an actual real-world increase of the prevalence. This could be explained by the fact that overall survival with psoriasis is rising, as Springate et al. found out to be true for the UK by comparing prevalence rates with incidence rates [8]. On the other hand, an increase in treatment frequencies is possible, too, as we derive prevalence rates from billing data. Here, the large number of newly developed treatment options for psoriasis over the last years could play a major role, so that more patients reach out for effective treatment.

### Strengths and limitations

The greatest strength of this study is its unique database, which represents approximately 90% of the German population. This offered the opportunity to analyse population-based trends on a small-scale regional level. The data cover five diseases for an eight-year period, which offers a profound base for comprehensive spatiotemporal analyses of prevalence development for widely spread diseases. In addition, to our knowledge, this study is the first to examine relations of comorbidities with psoriasis spatially. We were able to apply various statistical and geographical methods thoroughly to examine the regional and temporal patterns from different perspectives. Though, some limitations need to be addressed. To base the analysis on billing data has the disadvantage that only cases of psoriasis respectively comorbidities treated within the German SHI were documented. Thus, the treatment prevalence rather the population-based overall prevalence was assessed. In addition, there is no access to incidence data. It is therefore difficult to verify our assumptions about the increase in prevalence. We may also have underestimated the prevalence rates slightly by applying a case definition of physician consultations in at least two quarters within 12 months. This strategy might exclude mild cases, on the assumption that psoriasis in need of treatment would rather have a correlation to the development of a parallel existing comorbidity. Furthermore, it has the advantage to exclude misclassifications. It is highly recommended for the analysis of chronic diseases in secondary data analyses [51]. In addition, it must be mentioned that no clinical data are available and could therefore not be taken into account. This point is important because it is known that different clinical forms of psoriasis are associated with particular comorbidities. Finally, this is a retrospective study; statements about future (local) developments cannot be derived from the results.

## Conclusion

The results of the study show the first spatiotemporal association between psoriasis prevalence and comorbidities in Germany. This is particularly true for hypertension, diabetes and obesity. The relationship to affective disorders is less clear, as the spatial patterns differ from the patterns of psoriasis prevalence. The causalities must be analysed in more detail in order to be able to derive measures for improved care.
